# Design of Hybrid PAH Nanoadsorbents by Surface Functionalization of ZrO_2_ Nanoparticles with Phosphonic Acids

**DOI:** 10.3390/nano11040952

**Published:** 2021-04-08

**Authors:** Nadine Bou Orm, Thomas Gréa, Marwa Hamandi, Alexandre Lambert, Florent Lafay, Emmanuelle Vulliet, Stéphane Daniele

**Affiliations:** 1 College of Natural and Health Sciences, Zayed University, P. O. Box 144534 Abu Dhabi, United Arab Emirates; 2 CP2M-ESCPE Lyon, CNRS-UMR 5128, Université Claude Bernard Lyon 1, 43 Bd du 11 Novembre 1918, 69616 Villeurbanne, France; thomas.grea@gmail.com (T.G.); marwahmandi@gmail.com (M.H.); alexandre-lambert@live.fr (A.L.); 3 ISA, CNRS-UMR 5280, Université Claude Bernard Lyon 1, 5 rue de la Doua, 69100 Villeurbanne, France; Florent.LAFAY@isa-lyon.fr (F.L.); Emmanuelle.VULLIET@isa-lyon.fr (E.V.)

**Keywords:** ZrO_2_, nano-hybrids, adsorption, polycyclic aromatic hydrocarbons, surface grafting, phosphonic acids

## Abstract

This study focuses on the preparation of innovative nanocomposite materials based on surface modification of commercial nano-ZrO_2_ optimized from Brønsted acid–base surface reactions. This surface modification was carried out by direct grafting of suitable phosphonic acids bearing a vinylic or phenylic substituent in aqueous solution. Different loading quantities of the anchoring organophosphorus compounds were applied for each materials synthesis. The resulting nanohybrids were thoroughly characterized by infrared spectroscopy (DRIFT), solid-state nuclear magnetic resonance (NMR), nitrogen adsorption-desorption (BET), thermogravimetric analysis (TG), and X-ray photoelectron spectroscopy (XPS), demonstrating the reliability and efficient tunability of the surface functionalization based on the starting Zr/P ratio. Our nanocomposite materials exhibited a high specific surface area as well as complex porosity networks with well-defined meso-pore. The as-prepared materials were investigated for the adsorption of a mixture of 16 polycyclic aromatic hydrocarbons (PAHs) at 200 ng·mL^−1^ in an aqueous solution. Adsorption kinetics experiments of each individual material were carried out on the prepared PAHs standard solution for a contact time of up to 6 h. Pretreatments of the adsorption test samples were performed by solid-phase extraction (SPE), and the resulting samples were analyzed using an ultrasensitive GC-orbitrap-MS system. The pseudo-first-order and the pseudo-second-order models were used to determine the kinetic data. The adsorption kinetics were best described and fitted by the pseudo-second-order kinetic model. The correlation between the nature of the substituent (vinylic or phenylic) and the parameters characterizing the adsorption process were found. In addition, an increase of PAHs adsorption rates with phosphonic acid loading was observed.

## 1. Introduction

Polycyclic aromatic hydrocarbons (PAHs) are hydrophobic organic molecules that resulted from the condensation of at least two aromatic rings, being white or yellowish solids [1]. Their ubiquitous and persistent nature in the environment as well as their proven toxicity towards humans and ecosystems have raised general concern in recent years [2]. Hence, 16 of these chemicals were listed as priority pollutants by the US Environmental Protection Agency (US EPA) due to their common detection in the environment, their acute toxicity, and their ability to accurately represent the vast majority of the several hundred derivatives from their chemical class [3,4]. Their toxicity is mainly due to their carcinogenic properties, and it comes as well from their ability to suppress the immune system [5]. PAHs can occur from many anthropogenic or natural sources like forest fire, volcano eruption or vehicular emission, petroleum-related activity, storage, and transport [6]. The presence of these pollutants in surface water and soil mainly comes from the deposition of these airborne particles, which results in general pollution of PAHs in the environment [6]. Unfortunately, traditional wastewater treatments are not efficient enough to ensure their remediation in an aqueous environment [7]. Therefore, numerous methods have been proposed for the removal of PAHs from environmental matrices [8,9]. One of the most promising techniques is the adsorption method, which could have significant economic and environmental advantages over other proposed methods. PAHs removal by adsorption has been efficiently achieved with activated carbon, biochar, clay derivatives, but each of these proposed adsorbents implies significant drawbacks that need to be addressed prior to their democratization [10]. Among them, nanohybrid adsorbents have gained a lot of popularity during the last decades. These nano-adsorbents present interesting features that make them suitable candidates for PAHs remediation in the environment [11,12].

Different supports have been proposed, whether carbonaceous ones (outside the scope of this article) or natural [13] or manufactured inorganic supports functionalized on the surface or not [14,15]. ZrO_2_, because of its high chemical stability against corrosion and biocompatibility, has been shown to be highly suitable for applications involving the removal of organic pollutants from the environment [16,17,18]. Thus, unlike titania, which can undergo photocatalytic reaction leading to the degradation of the grafted organic part, zirconia is much less reactive under photoradiation [19]. In this regard, phosphonic acids (PAs) that cannot undergo homocondensation and that show a high affinity and stability for metal oxides have been widely studied for the surface modification of metal oxides such as titania, alumina, iron oxides, indium-tin-oxide (ITO), and zirconia [20,21,22,23,24]. In addition, PAs possess (like organosilicates) very stable P-C bonds to hydrolysis that provide a large variety of functional organic fragments available for functionalization. Finally, the high acid stability of the resulting hybrid has allowed to extend the field of application of these materials to complex oxidants and acidic matrices [24].

In this study, we report the synthesis and characterization of zirconia-based nanomaterials functionalized by phosphonic acids bearing a vinylic (VPA) or phenylic (PPA) substituent as nano-adsorbents suitable for PAH adsorption trough π–π interactions. The surface modification has been achieved by a simple approach of post-functionalization of commercial nanosuspensions to allow easy control of the final properties of the resulting nano-hybrids and take into account the economic prerequisites for possible integration in an industrial process. The grafting of the organic moieties has been achieved with different loading quantities to accurately assert the influence of the nature and the amount of the organic part on the synthesized materials’ resulting physicochemical properties. All materials were therefore fully characterized using a wide variety of characterization techniques (nitrogen adsorption-desorption (BET), infrared spectroscopy (DRIFT), solid state ^1^H ^31^P NMR, TGA,Differential Scanning Calorimetry (DSC) XPS, and elemental analysis) and then tested as an effective nanoadsorbent for the removal of 16 PAHs in aqueous solution (200 ppb); each sample being analyzed using a new ultra-sensitive gas chromatography (GC-orbitrap-MS) apparatus [25].

## 2. Materials and Methods

### 2.1. Chemicals and Reagents

All chemicals were of technical or analytical grade and used without further purification. Phenylphosphonic acid (PPA) (>98.0%, C_6_H_7_O_3_P, Mw = 158.09 g/mol) and Vinylphosphonic acid (VPA) (>95.0%, C_2_H_5_O_3_P, Mw = 108.03 g/mol) were purchased from TCI (Tokyo Chemical Industry). Zirconium (IV) oxide nanoparticles dispersion (99.9%; 10% by weight aqueous solution of a ZrO_2_ nanosuspension at pH 4–5) and ethyl acetate (C_4_H_8_O_2_, ACS reagent, >99.8%) were both of analytical grade and purchased from VWR. The 16 US EPA PAH mixture (500 µg/mL in Acetonitrile:Acetone:Toluene (6:3:1)) were purchased from LGC Standards. Deionized 18.2 MΩ·cm water was used throughout the whole experiment.

### 2.2. Syntheses of Surface Modified ZrO_2_ Nanoparticules by Phosphonic Acid

The general procedure is as follows: 12.33 g of the ZrO_2_ nanosuspension (10 mmol of ZrO_2_) were added to a 50 mL round-bottom flask. Different amounts of VPA solution diluted in distilled water or PPA powder (0.05, 0.1, 0.2, 0.4 mmol of VPA, respectively) were added slowly to the ZrO_2_ nanodispersion, and the mixture was stirred for 4 h at 100 °C. The molar ratio ZrO_2_/VPA and ZrO_2_/PPA ranged from 200:1 to 25:1. At the end of the reaction, the suspension obtained was centrifuged for 10 min at 12,000× *g* rpm. the solid phase was recovered then washed by adding 30 mL of distilled water, redispersing by sonication for 5 min, and then separating by centrifugation again. The washing procedure was repeated twice with water (2 × 30 mL). The white solid was then retrieved and dried in an oven at 95 °C for 24 h. The resulting pale-yellow solid was ground to a fine powder using a mortar and pestle and then stored in a labeled vial. The resulting materials were designated, respectively, by (ZrO_2_)*_x_*(VPA) and (ZrO_2_)*_x_*(PPA) where *x* = 200, 100, 50, 25 is the molar ratio related to the organic part grafted onto the inorganic support.

### 2.3. PAHs Batch Adsorption Test Procedure

Diluted solutions of the PAH mixture have been precisely conducted to assert the reproducibility of each test. An amount of 400 µL of the 16 US EPA PAHs mixture (500 µg·mL^−1^) contained in the commercial amber sealed bottles was collected using a micropipette and introduced into a 1 L volumetric flask containing 40 mL of absolute anhydrous ethanol. The resulting solution was manually shaken to obtain a homogeneous mixture and completed with distilled water to obtain a solution of 200 ng·mL^−1^ of PAH in water: ethanol (24:1) mixture. Adsorption tests for each material were achieved by introducing 10 mg of our synthesized nanoadsorbent (weighed on a precision balance) in a series of 125 mL flasks disposed on a multi-stirring platform with 100 mL of the 200 ng·mL^−1^ standard PAH solution with a stirring rate of 1000 rpm. For the kinetic study, each flask was taken out at a predefined time interval of 5, 15, 30 min and 1, 2, 4, 6 h and 20 mL samples of the resulting mixture were collected into centrifugation tubes. For the isotherm study, different PAH solutions were prepared in the same aqueous medium. The concentrations were varied from 50 to 400 ppb. An amount of 10 mg of ZrO_2_ or (ZrO_2_)_50_(PPA) was suspended in 100 mL of each PAH solution. After stirring the suspension at room temperature for 30 min at 300 rpm, the resulting mixture was collected into centrifugation tubes. The spent adsorbent particles were separated by centrifuging the collected samples at 12,000× *g* rpm for 10 min. An amount of 10 mL of the supernatant was introduced into a glass tube to be treated by solid-phase extraction (SPE) on commercial Strata-X 33u polymeric sorbent cartridges to extract non-adsorbed PAHs remaining in the sample from the aqueous matrix. An amount of 1 mL of isopropanol (10 vol% of the sample) was added to the tube containing the sample prior to SPE analysis, as our previous tests revealed that Heavy Molecular Weight (HMW) PAHs tend to be sorbed on the walls of the tube without any addition of organic solvents. The SPE procedure included three steps: conditioning the cartridge with successively 4 mL of ethyl acetate and 4 mL of 2.0 vol% isopropanol in water for removing substantial impurities present in the column and also activating the immobile phase; loading the column with the water sample at a controlled flow rate of 1–2 droplets per seconds (10 mL·min^−1^) followed by a drying step to remove moisture in the cartridge; and then collecting the analytes by eluting the cartridge with 8 mL of ethyl acetate. No preconcentration treatment was needed prior to Gas Chromatography-Mass Spectrometry (GC-MS) analysis since the analyzer system is sensitive enough to detect trace levels (ppb) [26]. Standard solutions of 5, 20, 50, 100, 150, and 200 ng·mL^−1^ were further elaborated to precisely calibrate the GC-orbitrap-MS system by diluting the starting 200 ng·mL^−1^ solution with water and extracting by SPE.

### 2.4. Equipment and Characterization

DRIFT experiments of our surface-modified nano-ZrO_2_ materials were carried out on a Nicolet 6700 FT-IR spectrophotometer (Thermo Fisher Scientific, Waltham, MA, USA) equipped with a Mercury Cadmium Telluride (MCT) detector (number of scans: 64, resolution: 4 cm^−1^). Nitrogen gas adsorption-desorption isotherms were carried out using a ASAP 2020 instrument (Micromeritics, Merignac, France) at 77 K based on the BET method for specific surface area measurement. Prior to analysis, solids were pretreated at 120 °C for 3 h with a thermal ramp of 10 °C·min^−1^ allowing their degasification. Determination of the organic grafting moieties of our nanohybrid materials were carried out by thermogravimetric analysis using TGA 2 STARe System (Mettler Toledo, Greifensee, Switzerland) with medium pressure alumina crucibles of 70µL (Ø 7 mm). A heating rate of 10 °C·min^−1^ was used from 25 °C to 900 °C under air atmosphere. Elemental analyses were carried out by ICP-OES Activa (Horiba Jobin Yvon, Longjumeau, France) in IRCELYON (Villeurbanne). 31P solid-state NMR spectra of our ZrO_2_ nanomaterials functionalized with phosphonic acid were obtained on a AVANCE III 300 MHz NMR spectrometer (Bruker, Billerica, MA, USA) with Magic Angle Spin (MAS) technique. Solid state MAS 31P NMR spectra were recorded using frequency of 121.4 MHz, at rotation speed of 10 KHz with a 4 mm ^15^N−^31^P/^1^H. The centrifugation of our suspension resulting from our synthesis and batch adsorption test were carried out using an 2.5-Liter Multilab centrifuge LISA (AFI centrifuge, Château-Gontier, France) equipped with an Angular Rotor 8x50–25° at rotation speed of 12,000× *g* rpm. Samples resulting from our batch adsorption tests were extracted using Rapid Trace SPE Workstation (Zymark)with commercial Strata X 33u polymeric sorbent cartridges (Phenomenex, Torrance, CA, USA). Analyses were carried out on a Trace 1310 GC equipped with a Triplus RSH autosampler and coupled to an Orbitrap-MS analyzer (Thermo Fisher Scientific, Waltham, MA, USA). All devices were from Thermo Fisher Scientific. A TG-5SilMS (Thermo Fisher Scientific, Waltham, MA, USA) column was used (30 m × 0.25 mm I.D, film thickness 0.25 µm) with helium as carrier gas (Helium flow: 1.2 mL/min). The injection was performed in splitless mode (1 µL) at a temperature of 325 °C with a split flow rate of 50 mL/min and splitless time of 0.8 min. The heating gradient was 25.0 °C/min to 240 °C; 5.0 °C/min to 280 °C; 25.0 °C/min to 325 °C (hold time: 8.40 min); and 25.0 °C/min to 350 °C for total running time of 27 min. Orbitrap-MS analyzer was employed using a 70 eV electronic impact set in full scan mode with a solvent delay of 3 min. The ion source and transfer line temperature were set to 250 °C. The scanned mass range was from 50 to 600 m/z.

### 2.5. Adsorption Kinetics

#### 2.5.1. Pseudo-First-Order Kinetic Equation

This well-known kinetic equation may be depicted as follows [27]:

(1) $$\frac{{\mathrm{dq}}_{t}}{\mathrm{dt}}={\;k}_{1}\;\left(q_{e}-q_{t}\right),$$

where q_e_ and q_t_ are the adsorption capacity of the adsorbent at equilibrium and at time t, respectively [mg/g]. k_1_ is the pseudo-first-order rate constant [min^−1^]. When integrating Equation (1), the linear form of the equation becomes:

(2) $$\;\ln\left({\;q}_{e}-q_{t}\right)={\mathrm{lnq}}_{e}-{\;k}_{1}t,$$

The adsorption rate constant, k_1_ [min^−1^], can be obtained from the slope of the linear plot of ln (q_e_ − q_t_) versus t.

#### 2.5.2. Pseudo-Second-Order Equation

The pseudo-second-order model can be expressed as follows [27]:

(3) $$\;\frac{{\mathrm{dq}}_{t}}{\mathrm{dt}}={\;k}_{2}\;{\left(q_{e}-{\;q}_{t}\right)}^{2},$$

where k_2_ is the pseudo-second-order rate constant [g/(mg min)]. On integration, this equation can be re-arranged to give the linear form:

(4) $$\;\frac{t}{q_{t}}=\;\frac{1}{k_{2}q2_{e\;}}+\;\frac{t}{q_{e}}\;,$$

The plot of t/q_t_ versus t gives a linear relationship, which allows the values of q_e_ and k_2_ to be computed.

### 2.6. Adsorption Isotherm

Adsorption isotherms measurements were carried out by varying the PAH molecules concentration between 50 and 400 ppb. All the experiments are carried out at room temperature for 30 min according to the kinetic study described in the previous paragraph. A reliable prediction of adsorption parameters can be obtained from equilibrium adsorption isotherm equations. In this study, the evaluation of the experimental results was based on Langmuir and Freundlich isotherms models. Langmuir [28] isotherm supposes monolayer adsorption onto a surface containing a finite number of identical and uniform adsorption sites with no transmigration of adsorbate in the plane of the surface and no interaction between the adsorbed molecules on the adsorbent surface. The linearized Langmuir isotherm equation is given as:

(5) $$\frac{1}{q_{e}}=\;\frac{1}{q_{m}}\;+\;\frac{1}{q_{m}K_{L}C_{e}}$$

where q_e_ and C_e_ are the adsorption capacity (mg g^−1^) and the PAH concentration (mg L^−1^) at equilibrium, respectively, qm and K_L_ represent the maximum adsorption capacity of adsorbents (mg g^−1^), and the Langmuir adsorption constant (L mg^−1^), respectively. The Freundlich [29] isotherm is an empirical equation. It is another approach that is used for the description of the multilayer and heterogeneous adsorption of molecules to the adsorbent surface. It is expressed by the following linear form:

(6) $${\mathrm{lnq}}_{e}={\;\mathrm{lnK}}_{F}+\;\frac{1}{n_{F}}{\;\mathrm{lnC}}_{e}$$

where K_F_ and n_F_ are Freundlich isotherm constants. K_F_ (mg·g^−1^) indicates the adsorption capacity of the adsorbent toward the adsorbate and n is an indicator for the degree of the surface heterogeneity and describes the distribution of the adsorbed molecules on the adsorbent surface. A value of higher n reflects the higher intensity of adsorption.

## 3. Results

### 3.1. Characterization

Commercial ZrO_2_ nanosuspension (10 wt% in H_2_O, pH 4–5, dp < 100 nm) purchased from Sigma Aldrich (Wassergasse, Switzerland) was previously characterized with XRD powder diffractograms in literature and this analysis confirmed the presence of a mixture of tetragonal (~58%) and monoclinic (~42%) ZrO_2_ [24]. The commercial ZrO_2_ nanosuspension was also thoroughly described in the same study with TEM imaging, revealing that the sample was rather polydispersed in size (20 ± 10 nm). The XRD powder diffractograms and TEM images obtained during this study revealed that the grafting process did not modify the morphology and structure of the ZrO_2_ starting support.

**Figure A1:**
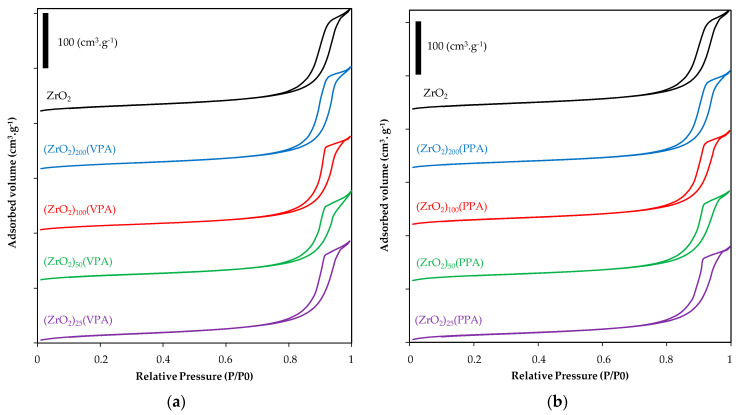
Isotherms of (**a**) (ZrO_2_)*_x_*(VPA) and (**b**) (ZrO_2_)*_x_*(VPA) Samples (Zr/P ratio (*x*) = 25, 50, 100, 200) and bare ZrO_2_.

Nitrogen adsorption-desorption isotherms spectra for our synthesized materials and bare ZrO_2_ are shown in Figure A1 and recorded after the degasification of our compounds at 120 °C for 3 h. All of the resulting nanomaterials exhibited almost identical isotherms to the starting material. All compounds without discrimination displayed type IV(a) isotherms based on the IUPAC terminology [30], representing mesoporous adsorbents where pore condensation occurs just after the initial monolayer-multilayer adsorption on the mesopore walls. Our isotherms exhibit a final inflection point instead of a simple saturation plateau that reveals the complex pore network of our materials. Furthermore, the nanohybrids isotherm presents an H2(b)-type hysteresis loop, which further highlights the complex pore structure in which the network effects are significant. Hysteresis loops generally occur during multilayer physisorption and are usually associated with capillary condensation resulting from either adsorption metastability and/or pore blocking effects. In our case, the H2(b)-type hysteresis loop seems to support the hypothesis of very complex pore networks in our nanomaterials. The isotherm study agrees with the pore size measurement based on the BET method, revealing that all our materials are mesoporous, which is beneficial for our PAH adsorption application. However, the major pore’s width distribution seems to be shifted from the starting material, revealing the pore structure’s interparticle origin that is affected by the surface functionalization of the starting ZrO_2_ support. As shown on Table A1, the specific surface areas of the synthesized materials are in the same range as the starting material (49–57 m^2^·g^−1^), suggesting that our grafting process did not affect the morphology of the inorganic support.

**Figure 1:**
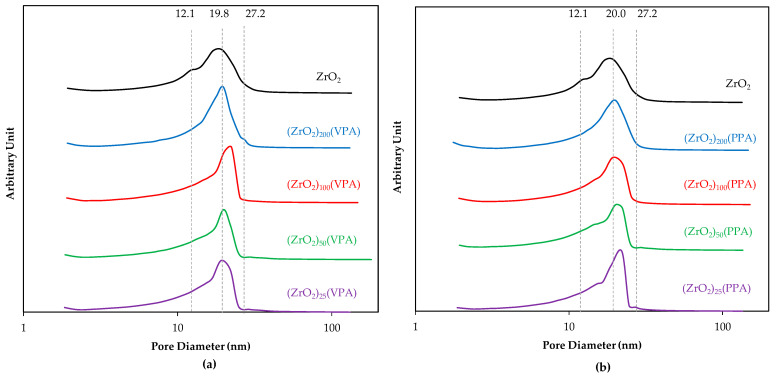
BJH pore size distributions for (**a**) (ZrO_2_)*_x_*(vinylic (VPA)) and (**b**) (ZrO_2_)*_x_*(phenylic (PPA)) samples (Zr/P ratio (*x*) = 25, 50, 100, 200) and bare ZrO_2_.

Furthermore, Barrett, Joyner, and Halenda (BJH) pore distribution exhibited in Figure 1 revealed that pore structure was slightly changed in the case of ZrO_2_-VPA materials but kept in the polydispersed range of 20 ± 10 nm, being consistent with the result of the previous study [24]. However, for PPA derivatives, the major pore size distribution shifts as a function of the Zr/P ratio with a linear increase in pore width for x = 25, a trend that was not revealed by the BET analysis of the VPA derivatives compounds. Since pore structure is defined as intergranular porosity for our nanomaterials, pore width seems to be directly related to the nature of grafting moieties. This is also attested by the isotherm spectra of our PPA materials showing desorption branches of the hysteresis tending towards a rather steep desorption branch, a characteristic of H2(a) loops for highly loaded materials. This observation results from a pore-blocking phenomenon in a narrow range of pore necks, which can be explained by the size of phenyl substituents being wider than vinyl ones [31].

**Figure 2:**
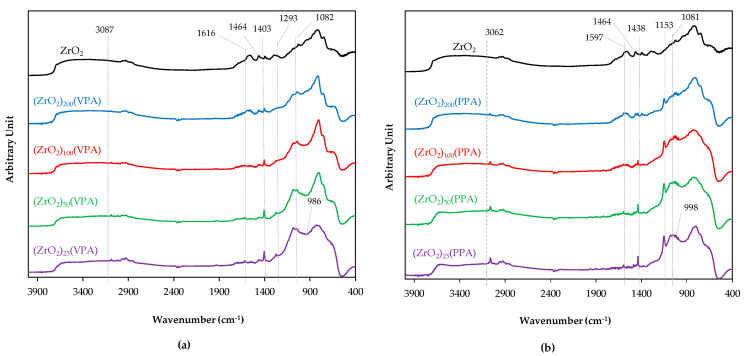
Infrared spectroscopy (DRIFT) spectra of (**a**) (ZrO_2_)*_x_*(VPA) and (**b**) (ZrO_2_)*_x_*(PPA) samples (Zr/P ratio (*x*) = 25, 50, 100, 200) and bare ZrO_2_.

Characterization of the effectiveness of the surface functionalization of our nanohybrid materials has first been carried out using DRIFT analysis, since this technique allows the direct analysis of our powders without prior preparation and result in brief analysis where the data are collected on the overall sample. Both ZrO_2_-VPA and ZrO_2_-PPA spectra, shown in Figure 2, were recorded in absorbance to clearly emphasize on representative peak of the chemical transformation between bare ZrO_2_ and the resulting ZrO_2_-VPA and ZrO_2_-PPA nanohybrids.

All DRIFT spectra exhibited the same bands regardless of the Zr/P ratio of the materials. Strong absorption bands around 800 and 1464 cm^−1^ can be easily ascribed to the Zr-O bond since these bands are exhibited in both the bare ZrO_2_ and ZrO_2_-VPA or ZrO_2_-PPA spectra. This assignment was further supported on these ZrO_2_-PPA spectra, since we observed a clear linear disappearance of the Zr–O band at 1464 cm^−1^ as a function of the increase in the Zr/P ratio. In addition, the broad band centered at 3300 cm^−1^ is characteristic of the O-H bond and can be attributed here to surface hydroxyl for bare zirconia and to synthetic solvents. The P–O stretching peak, at 986 cm^−1^ for ZrO_2_-VPA, was slightly shifted to 998 cm^−1^ for ZrO_2_-PPA materials and was even more covered by the characteristic aromatic absorption occurring in the fingerprint between ~1000 and ~1100 cm^−1^ [31]. The usual characteristic weak to medium absorption band of P–O–H located near 2600 and 2200 cm^−1^ was not displayed on our spectra, which may be due to the low concentration of phosphonic acid in our samples. Same observations were made for TiO_2_-VPA materials [24]. In the case of ZrO_2_-VPA spectra, the 3087 cm^−1^ band results in the adsorption of the C–H stretching vibration of the vinylic C sp^2^, represented by the adsorption peak of the C=C stretching vibration at 1616 cm^−1^. In the case of ZrO_2_-PPA spectra, the medium absorption peak at 3062 cm^−1^ results from the C–H stretching vibration of the aromatic function, further evidenced by the weak adsorption band of the C=C stretching vibration occurring at 1597 cm^−1^. The medium absorption peak at 1438 cm^−1^ was characteristic and resulted from the P–C bond’s vibration linked to an aromatic substituent. Moreover, the band intensity linearly increases with the Zr/P ratio of our composite materials, supporting the evidence that our grafting was efficiently achieved. It is worth noting that this band’s absorption frequency was shifted based on the substituent bearing the carbon linked to the phosphorus; 1403 cm^−1^ for the vinyl and 1438 cm^−1^ for the phenyl. As with ZrO_2_-VPA, ZrO_2_-PPA compounds exhibited the absorption of the vibrational stretching band of O–P–O found at 1081 cm^−1,^ which is still moderately covered by the zirconia absorption area. Furthermore, characteristic P=O stretching vibration was also retrieved in the case of ZrO_2_-PPA materials but significantly shifted at 1153 cm^−1^, whereas it was found at 1273 cm^−1^ for ZrO_2_-VPA samples, which can induce a different binding conformation between the two organophosphorus compounds in regard to zirconia surface modification since this bond has been reported to be very sensitive to association effects [32]. The remaining band at 1273 cm^−1^ is highly characteristic of the P=O stretching vibration, which is almost independent of the type of compound or the size of the substituents and can easily help us address the grafting efficiency. In addition, the presence of a P=O function for our material induces that the grafting occurs preferentially by the reaction of the P-OH function on surface hydroxyls, whether they are monocoordinated or bicoordinated. Finally, the intensity of peaks ascribed to phosphonic acid grafting was significantly and linearly shifted based on the Zr/P ratio, which could quantitatively address the grafting quantity present in the final material.

**Figure 3:**
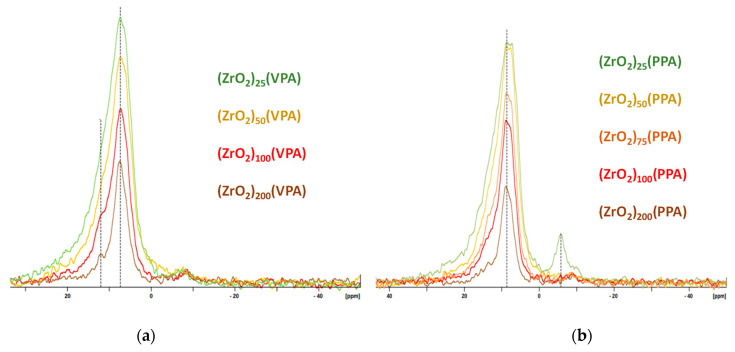
^31^P CP MAS NMR spectra of (**a**) (ZrO_2_)*_x_*(VPA) and (**b**) (ZrO_2_)*_x_*(PPA) samples (Zr/P ratio (*x*) = 25–200).

Solid state ^31^P NMR has been used to better apprehend the coordination of the anchoring group onto inorganic support thanks to a wide variety of observed nuclei in organic grafting moieties. The solid-state ^31^P MAS NMR spectra of our ZrO_2_-VPA and ZrO_2_-PPA samples are given in Figure 3. In the case of ZrO_2_-VPA, all our materials spectra displayed a distinct resonance downfield shift at 7.4 ppm and broadened in regard of the starting vinylphsophonic acid reagent, which, according to the literature, gave a sharp peak at 18 ppm [33]. This downfield shift underlines the fact that the grafting of the vinylphosphonic acid was correctly achieved on the ZrO_2_ surface. Moreover, the signal’s broad characteristic results in the sensitivity of the analysis to the variation of bond angles; therefore, coordination sites with slightly distorted geometries induce many different peaks that result in a broad signal covering each of them [19,20,21,22,23,24]. Studies with ^31^P NMR have shown that the predominant binding state for monophosphonic acids on zirconia surface was generally bidentate and resulted in a peak at around 7 ppm, which correlates with our ^31^P NMR study and in good agreement to our DRIFT data that revealed P=O stretching vibration. Furthermore, a well-defined peak appeared on the (ZrO_2_)_200_(VPA) spectrum at around 12 ppm, which seems to be induced by a tridentate coordination mode based on an identical study conducted on surface-modified nano-TiO_2_ by VPA [31]. In the case of ZrO_2_-PPA, the spectra displayed a distinct downfield resonance shift at 8.7 ppm and broadened in comparison to the bare phenylphsophonic acid reagent that was reported in the literature to give a precise, sharp peak at 20.8 ppm [33]. The observed shift between our VPA and PPA materials is in the same range of those reported in the literature [24]. For the most PPA-loaded samples, a distinct downfield peak at −5.60 ppm reveals molecular zirconium phosphonate presence and the corrosion of ZrO_2_, which could be explained by the addition of pure non-solubilized PPA [34].

**Figure 4:**
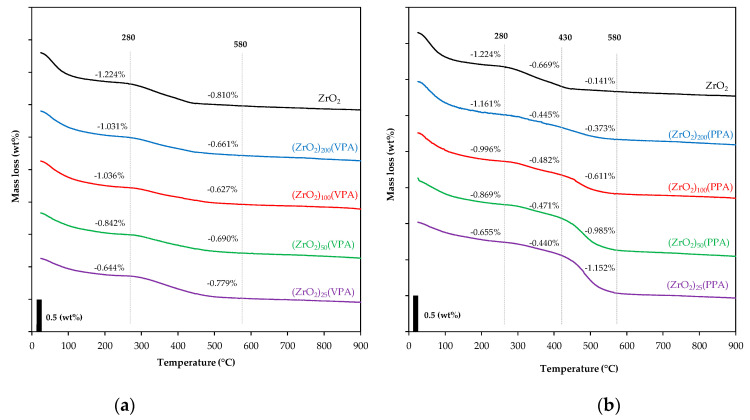
Thermogravimetric analysis of (**a**) (ZrO_2_)*_x_*(VPA) and (**b**) (ZrO_2_)*_x_*(PPA) samples (Zr/P ratio (*x*) = 25, 50, 100, 200) and bare ZrO_2_ in the range of 25–900 °C.

**Figure A2:**
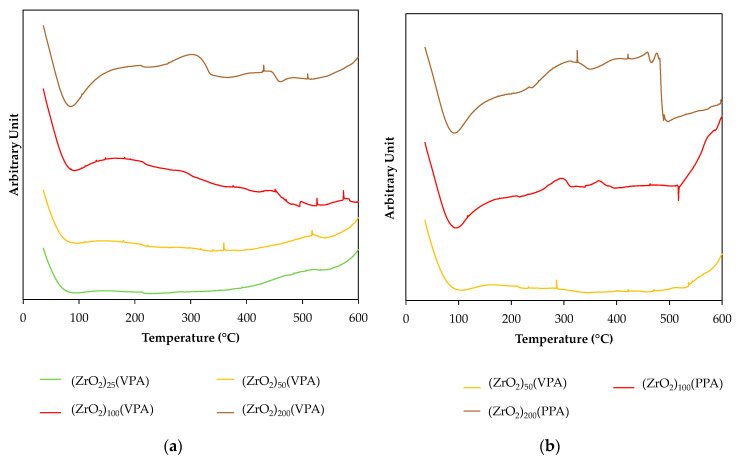
Differential Scanning Calorimetry analysis of (**a**) (ZrO_2_)*_x_*(VPA) and (**b**) (ZrO_2_)*_x_*(VPA) Samples (Zr/P ratio (*x*) = 50, 75, 100, 200) in the range of 25–600 °C.

**Figure A3:**
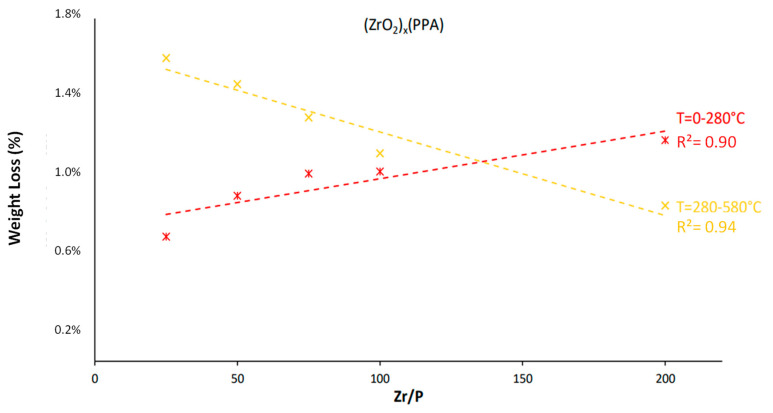
Variation of organic weight loss in the range of 0–280 °C and 280–580 °C in thermogravimetric analysis as a function of the starting Zr/P ratio for (ZrO_2_)*_x_*(PPA) Samples.

TGA experiments were carried out on all synthesized ZrO_2_-VPA and ZrO_2_-PPA materials to quantitatively estimate the VPA and the PPA content. Figure 4 shows the thermogravimetric analysis recorded under air from 25 °C to 900 °C with a thermal ramp of 10 °C/min. In the case of ZrO_2_-VPA, all curves displayed two defined major mass losses, one from 25 °C to 280 °C and the other from 280 °C to 580 °C. In the case of ZrO_2_-PPA, TGA curves reveal three major mass losses step ranging from 25 °C to 280 °C, 280 °C to 430°C, and 430 °C to 580 °C, respectively. The first major mass loss for both ZrO_2_-VPA and ZrO_2_-PPA was ascribed to the evaporation of remaining water and solvents in the samples, further evidenced by the endothermic step appearing on the DSC analysis conducted afterward for this range of temperature (Figure A2). For ZrO_2_-VPA, the second mass loss was due to the combustion of grafted surface vinyl phosphonate as well as the dehydration of the surface hydroxyl groups of the zirconia. The mass loss evolution was in agreement with the starting Zr/P ratio of the materials since the most concentrated organic material shows the highest mass loss in this temperature range. DSC experiments were carried out to try to precisely attribute the different temperature ranges where the surface hydroxyl and PAs were degraded. Based on our study conducted for TiO_2_ [23], DSC should have revealed two distinct exothermic peaks that could have helped us determine each mass loss and efficiently measure our nanohybrids’ organic part. However, in our case, the organic moiety quantity was too low to induce a well-defined DSC curve for our materials, even for the most concentrated one (ZrO_2_)_25_(VPA). Unlike VPA derivatives, our ZrO_2_-PPA compounds have presented two distinct mass losses in the range of 280 °C to 580 °C. The mass loss occurring from 280 °C to 430 °C has been attributed to the combustion of the remaining surface hydroxyl function after surface modification, whereas the one from 430 °C to 580 °C was clearly assigned to the organic grafting degradation since the mass loss quantity is linearly proportional to the initial Zr/P ratio of the considered materials. By plotting the mass loss between 25 and 280 °C and that between 280 and 580 °C as a function of the theoretical Zr/P ratios (Figure A3), we can efficiently demonstrate that the mass losses occurring followed a linear trend with a high regression coefficient of 0.90 and 0.94, respectively, for the first and combined second and third mass losses. Nevertheless, these mass losses cannot be used to accurately calculate the number of organic moieties present on the surface of our materials since phosphate residues are still present after calcination at 900 °C as shown by previous TGA studies on similar materials [24]. Thus, trying to quantify the grafting part with given TGA mass losses efficiently would lead to an underestimated value of the organic moieties present in the nanohybrid.

In order to accurately determine the Zr/P ratio of our nanomaterials, elemental analyses were carried out on the most representative samples of the VPA and PPA-based derivatives. The elemental analyses confirmed the grafting of a quantity of P (Table 1) in adequacy with the initial ratios in the case of ZrO_2_-VPA. However, in the case of ZrO_2_-PPA, they showed lower than theoretical amounts of P, which could confirm that some of the PPA were eliminated as molecular species such as phosphonates observed in ^31^P NMR.

**Figure A4:**
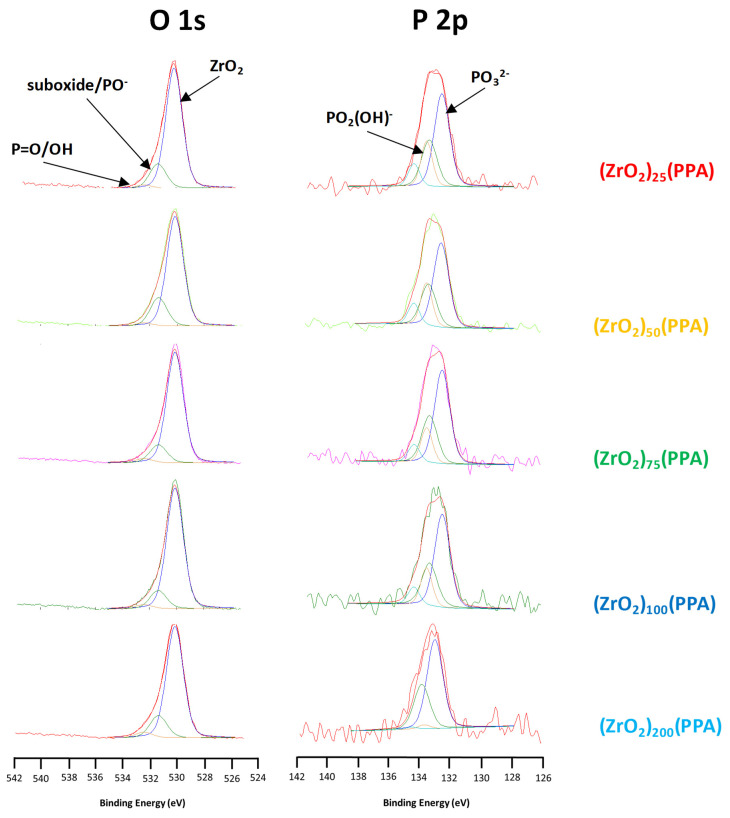
X-Ray Photoelectron Spectroscopy study of (ZrO_2_)*_x_*(PAA) Samples (Zr/P ratio (*x*) = 25, 50, 100, 200).

**Figure A5:**
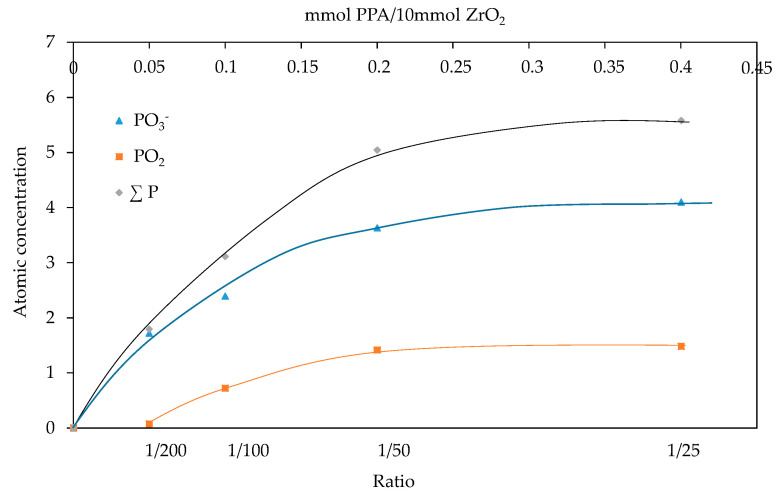
Variation of the surface species content of ZrO_2_ as a function of the ratio used, according to X-ray Photoelectron Spectroscopy study of (ZrO_2_)*_x_*(PAA) Samples (Zr/P ratio (*x*) = 25, 50, 100, 200).

The XPS study was carried out to match the observation of our solid NMR analysis and to accurately describe the surface coordination mode of our phosphonic acids on our nanozirconia, based on a study that examined the use of XPS to efficiently discriminate the binding modes of PPA on ZrO_2_ [35]. XPS studies have been carried out on our (ZrO_2_)*_x_*PPA materials (Figure A4). They have allowed us to confirm that the total amount of phosphorus detected in XPS increases as the Zr/P ratio of our materials increases, proving once again that our approach is effective (Table 1). Finally, it would seem that the majority of the PPA was grafted according to a tridentate mode and no monodentate mode (Figure A5).

### 3.2. Adsorption Efficiency and Kinetic Study

Adsorption kinetics studies have been conducted to determine the extraction efficiency and the selectivity of our (ZrO_2_)*_x_*(VPA) and (ZrO_2_)*_x_*(PPA) nanomaterials towards the targeted PAHs. These tests have been carried out with standard aqueous solutions of the 16 US EPA PAHs at 200 ng·mL^−1^ (ppb) for different contact times of 5, 15, 30, 60, 120, 240, and 360 min with 1000 rpm stirring. Adsorption tests have shown an efficiency higher than 90% for all PAHs. In general, the adsorption equilibrium was reached within 30 min of contact without saturation of the nanoadsorbent, regardless of the Zr/P starting ratio, since extraction percentages were found in the same range for each material after 6 h of contact.

The kinetic parameters of PAHs adsorption by our adsorbents were determined by applying the pseudo first-order model (Equation (2)) and the pseudo second-order model (Equation (4)), respectively, to the experimental data. It was found that the values of the q_e_ calculated from the pseudo first-order model did not fit the corresponding experimental q_e_ values. In addition, the correlation coefficient (R^2^) values determined for this model were less than 0.85. On the other hand, the pseudo second-order model’s application led to similar experimental and calculated q_e_ values, as well as R^2^ values that were within the range 0.95–0.99. This result indicates that the PAH adsorption mechanism is better explained in terms of the pseudo second-order reaction model. The main assumptions of this kinetic model are the following:

Adsorption occurs only at specific binding sites located on the surface of the adsorbent.

The adsorption energy does not depend on the formation of a layer on the adsorbent surface.

The second-order equation governs the rate of the adsorption process.

No interaction occurs between the molecules adsorbed on the surface of the adsorbent.

**Figure A6:**
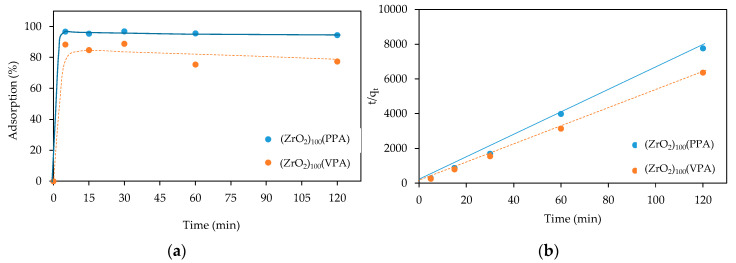
(**a**) Evolution with time of benz(a)anthracene adsorption using (ZrO_2_)_100_(VPA) and (ZrO_2_)_100_(PPA) and (**b**) Pseudo-second-order kinetic plots for the adsorption of benz(a)anthracene using (ZrO_2_)_100_(VPA) and (ZrO_2_)_100_(PPA).

Figure A6 shows an example of applying the pseudo-second-order model to the data in the form of t/q_t_ versus t plots. These were linear in all cases, allowing the values of k_2_ and q_e_ to be calculated from the corresponding slopes and intercepts. All kinetic parameters obtained using pseudo-second-order models are listed in Table 2.

**Figure 5:**
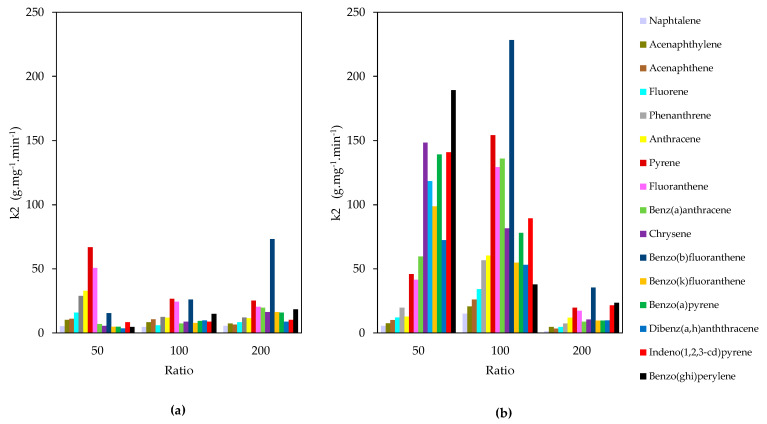
Pseudo-second order rate constant of PAHs adsorption by (**a**) (ZrO_2_)*x*(VPA) and (**b**) (ZrO_2_)*_x_*(PPA) samples (Zr/P ratio (*x*) = 50, 100, 200).

**Figure A7:**
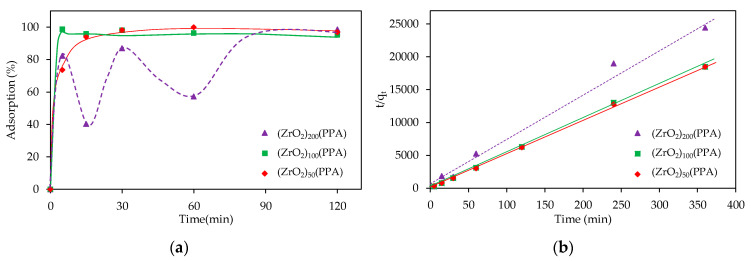
(**a**) Evolution with time of benzo(a)pyrene adsorption using (ZrO_2_)*_x_*(PPA) Samples (Zr/P ratio (*x*) = 50, 100, 200) and (**b**) Pseudo-second-order kinetic plots for the adsorption of benzo(a)pyrene using (ZrO_2_)*_x_*(PPA) Samples (Zr/P ratio (*x*) = 50, 100, 200).

Although the surface loading of ZrO_2_-PPA is low compared to VPA (Table 2), all (ZrO_2_)*_x_*PPA nanomaterials displayed better affinities to PAHs than (ZrO_2_)*_x_*VPA with adsorption rates between 50 and 230 g·mg^−1^·min^−1^ % for Heavy Molecular Weight (HMW) PAHs (Figure 5), whereas the adsorption rate constant of ZrO_2_-VPA was in the range of 10–70 g·mg^−1^·min^−1^. Figure A6 shows the adsorption of benz(a)anthracene using (ZrO_2_)_100_(PPA) or (ZrO_2_)_100_(VPA). (ZrO_2_)_100_(PPA) showed 97% of adsorption during the first 5 min of the test compared to (ZrO_2_)_100_(VPA), which showed 85% of adsorption during the same short period. In addition, (ZrO_2_)_50_(PPA) and (ZrO_2_)_100_(PPA) showed higher adsorption rates than (ZrO_2_)_200_(VPA), revealing some influence of the organic charge on the kinetic part of the adsorption. Figure A7 shows the adsorption curves of benzo(a)pyrene using (ZrO_2_)_50_(PPA), (ZrO_2_)_100_(PPA), and (ZrO_2_)_200_(PPA). Adsorption curves showed that a higher PPA content resulted in a more stable system with a higher adsorption capacity.

Furthermore, adsorption rate constants presented in Figure 5 showed competitive adsorption between Light Molecular Weight (LMW) and Heavy Molecular Weight (HMW) PAHs, which is more significant in the case of ZrO_2_-PPA nano adsorbents. HMW PAHs have more significant affinities to the adsorbent surface than LMW PAHs which have an adsorption rate constant less than 30 g·mg^−1^·min^−1^ in both ZrO_2_-PPA and ZrO_2_-VPA. However, for adsorption capacities, both showed similar values in the range of 14.5–19.5 µg·g^−1^.

In general, there is little difference in the case of VPA, both in terms of capacity and affinity. However, in the case of PPA, capacity remains almost unchanged, but affinity is increased by increasing the graft content to reach a pseudo-plateau from a ratio greater than 1/100. This behavior can be correlated with phosphorus species’ content on the surface of ZrO_2_ determined by XPS. As shown in Figure A5, the amount of PO_3_^-^ and PO_2_^-^ on the ZrO_2_ surface increased with the amount of PPA added until a plateau was reached. It started between the ratio 1/100 and the ratio 1/50.

**Figure 6:**
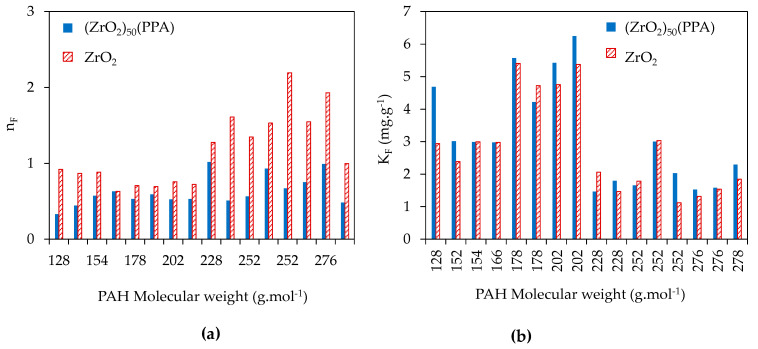
Variation of Freundlich parameters: (**a**) nF and (**b**) KF as a function of molecular weight of the 16 PAH molecules.

Isotherm parameters studied for ZrO_2_ and (ZrO_2_)_50_(PPA) were obtained by fitting the adsorption equilibrium data to the isotherm models, and are listed in Table A3. It can be noticed that the adsorption isotherms are fitted better by the Freundlich model since the Langmuir parameters had negative values, suggesting that PAH adsorption onto ZrO_2_ and (ZrO_2_)_50_(PPA) is a heterogeneous coverage. The correlation coefficient (R^2^) values for the Freundlich model are higher than 0.95. Figure 6 presents the variation of Freundlich parameters as a function of molecular weight of the 16 PAH molecules.

## 4. Discussion

Phosphonic acids (PA) R-PO(OH)_2_ with R = vinyl (VPA), phenyl (PPA) were chosen in this study because they condense efficiently and selectively with the surface hydroxyl groups of the metal oxide, leading to the formation of a monolayer of unsaturated ligand and very stable ionocovalent P–O–M bonds [34]. VPA or PPA grafting was based on acid–base Brønsted surface reactions. This reaction was carried out at 100 °C for 4 h in aqueous solution, allowing the surface modifications of ZrO_2_ nanoparticles and thus allowing to obtain inorganic–organic hybrid nanomaterials (as a white powders). The commercial ZrO_2_ nanosuspension’s pH was set around 4–5 to avoid the support aggregation if it gets close to the isoelectric point (IEP) of zirconia (around 9) [24].

**Figure 7:**
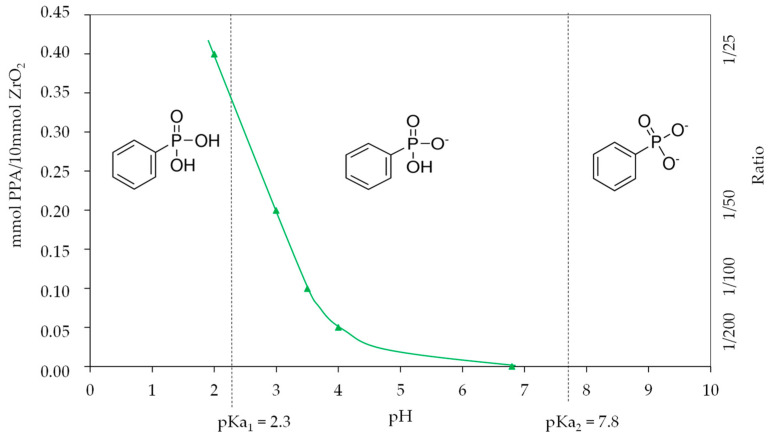
pH variation as a function of the concentration of PPA in solution and predominance of phenylphosphonate species as a function of pH.

Depending on the yield of PPA added to this suspension, the pH can vary, as shown in Figure 7. By considering the PPA species present in the suspension under different pH conditions, the mechanism by which PPA acts can be understood.

The pKa values for PPA are 2.3 and 7.8 [36,37]. The deprotonation equilibria for PPA are as follows:

Ph–PO(OH)_2_ (aq) ↔ Ph–PO_2_^−^ (OH) (aq) + H^+^(aq) (pKa1 = 2.3) [36]

Ph–PO_2_^−^ (OH) (aq) ↔ Ph–PO_3_^−^ (aq) + H^+^(aq) (pKa2 = 7.8) [36,37]

In the pH range of the resulting suspension, the dominant species of PPA are Ph-PO_2_^−^ (OH). In this case, it can easily react with the ZrO_2_ surface, forming a bidentate coordination. As the isoelectric point of zirconia is around 9, the coordination mode can evolve from bidentate to tridentate coordination as found by XPS and NMR analysis. It is important to note that at a relatively high PPA amount (ratio 1/25), the dominant species is neutral PPA, which can lead to corrosion of ZrO_2_ and can form zirconium phosphonate as shown by NMR analysis.

Furthermore, it is worth noting that the substitution of the hydrophilic surface hydroxyls of the metal oxide with PA bearing an unsaturated hydrophobic function resulted in rapid precipitation of the synthesized nanohybrid for the materials with the highest organic charge. Additionally, this phenomenon was observed more for phenyl PA derivatives than vinyl PA because the aromatic function has a lower aqueous solubility than simple unsaturation.

The hydrophobic aspect of the aromatic function in the case of PPA allows having more affinity to the PAHs molecules compared to VPA as determined by the kinetic study carried out for the two series of nanomaterials containing PPA or VPA. Indeed, the kinetic study has shown that, unlike VPA, in the PPA grafting and thanks to the aromatic ring’s hydrophobicity, the resulting nanomaterials have more affinity to the PAH molecules, especially for the heavier ones. This grafting did not change the nanomaterials’ textural properties; therefore, no difference was observed in terms of adsorption capacity in both cases of VPA and PPA.

**Figure 8:**
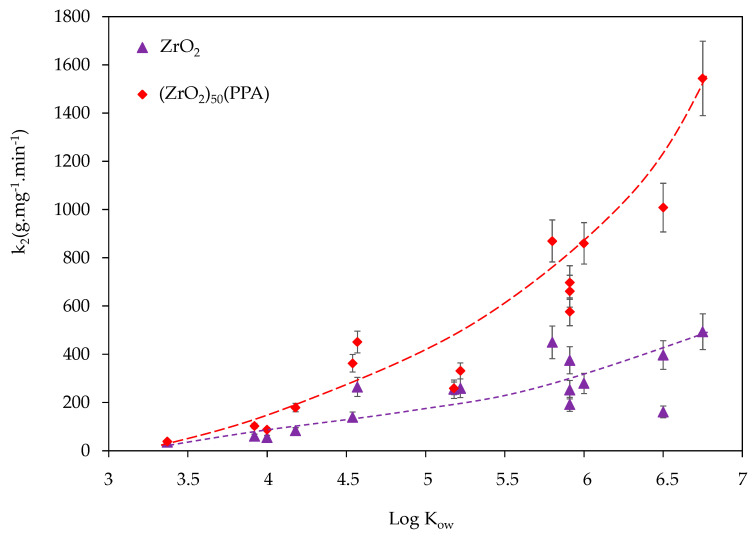
Variation of adsorption constant rate (k_2_) as a function of octanol–water partition coefficient (Kow) for the 16 PAH molecules in the case of ZrO_2_ or (ZrO_2_)_50_(PPA).

The properties of organic contaminants also affect the adsorption process. The hydrophobicity and hydrophilicity, the surface charge, and the functional groups of the pollutants can affect their adsorption behaviour. In the case of PAHs molecules, only the hydrophobicity and hydrophilicity could be a factor affecting the adsorption process. The octanol–water partition coefficient (Kow) is a useful physiochemical property to model the hydrophobicity and hydrophilicity of organic pollutants generally and PAH specifically. Figure 8 presents the variation of adsorption constant rate (k_2_) as a function of octanol–water partition coefficient (K_ow_) for the 16 PAH molecules in the case of ZrO_2_ or (ZrO_2_)_50_(PPA) tested at room temperature with a stirring rate of 300 rpm and an initial PAH concentration of 200 ppb. As shown in Figure 8 (and Table A2), the adsorption rate constant k_2_ is increasing in the same order as their octanol–water partition coefficients, which demonstrate that hydrophobic interaction can be a main mechanism for the adsorption process [38].

According to the variation of Freundlich parameters presented in Figure 6, the constant K_F_ related to the adsorption capacity does not present notable variation between ZrO_2_ and (ZrO_2_)_50_(PPA), which is correlated with the kinetic study revealing a constant q_e_ almost unchanged between grafted and ungrafted ZrO_2_. However, the values of n_F_ are quite different between ZrO_2_ and (ZrO_2_)_50_(PPA), showing a different adsorption mechanism. Ungrafted ZrO_2_ is a polar species while PAHs are rather nonpolar molecules. In this case, the interaction that can form is probably the inductive force, also called Debye interaction. The values of n_F_ increase as a function of the size of the PAH molecules. Indeed, the interaction is stronger since it increases with the polarizability, knowing that the polarizability rises with the size of the molecules, which clearly shows that the types of interaction between the ungrafted ZrO_2_ and the PAHs are indeed Debye interactions.

After surface functionalization by PPA, the polarity of the material surface decreases since phenyl groups are apolar. Thus, the interaction between the PAH molecules and the phenyl groups grafted on the surface of ZrO_2_ is an instantaneous dipole interaction, also called dispersion forces or London interaction. The dissociation energy of London interaction is less than 1 kcal.mol^−1^, which is coherent with n_F_ having values less than 1 in the case of (ZrO_2_)_50_(PPA). This low energy interaction would facilitate the regeneration of the material for reuse.

## 5. Conclusions

In conclusion, a straightforward and eco-friendly approach for surface modification of commercial ZrO_2_ nanosuspension was developed to precisely tune the inorganic support’s surface functionalization without altering its structural and textural properties. Simple phosphonic acids bearing vinylic or phenylic substituents were used as a suitable anchoring group with a high affinity toward zirconia surface modification. The innovativedesigned nanocomposite materials were elaborated bearing different amounts of the organic grafting part to address the influence of their loading amount on the resulting nanohybrids materials’ structural and physicochemical properties. Further on, each material was thoroughly described with a wide variety of characterization: DRIFT, ^1^H ^31^P solid NMR, TGA, DSC, BET, XPS, and elemental analysis, to precisely define their different specific properties (high specific surface area, unique mesoporous networks, and various binding mode of the anchoring group). The synthesized nanomaterials were then used as nanoadsorbent to remediate the alarming PAHs pollution occurring worldwide and in numerous environmental matrices. Kinetics adsorption studies of each material towards a prepared standard aqueous solution containing trace levels of the 16 US EPA PAHs were carried out and showed that our systems are highly efficient. The application of various kinetic models to the adsorption data indicated that PAHs’ adsorption was best explained in terms of a pseudo-second-order kinetic mechanism. The adsorption kinetics parameters for the adsorption process have been investigated, indicating competitive adsorption between LMW and HMW PAHs, the higher affinity of ZrO_2_-PPA compared to ZrO_2_-VPA towards, and some influence of the organic charge towards the kinetic part of the adsorption.

The samples’ pretreatments were ensured by solid-phase extraction using commercial cartridges prior to their analyses by a novel and ultrasensitive GC-orbitrap-MS system that has recently shown high detection features toward PAHs trace levels in aqueous solution. This study lay the foundation of zirconia-based nanocomposite materials as highly efficient and eco-friendly adsorbents to address PAHs ubiquitous pollution.

## Figures and Tables

**Table 1:** Quantitative analysis of (ZrO_2_)*_x_*(VPA) and (ZrO_2_)*_x_*(PPA) nanocomposites using XPS and elemental chemical analysis. (*** = not done)

	Elemental Chemical Analysis			XPS		
Adsorbent	Atomic Ratio Zr/P		Measured P (wt%)	PO_3_^-^	PO_2_(OH)	∑P
	Theoretical	Measured				
(ZrO_2_)_200_(PPA)	200	***	***	1.72	0.07	1.79
(ZrO_2_)_100_(PPA)	100	300	0.08	2.39	0.72	3.11
(ZrO_2_)_50_(PPA)	50	122	0.19	3.63	1.41	5.04
(ZrO_2_)_25_(PPA)	25	***	***	4.10	1.48	5.58
(ZrO_2_)_100_(VPA)	100	77	0.31	***	***	***
(ZrO_2_)_50_(VPA)	50	47	0.5	***	***	***

**Table 2:** Adsorption capacities (q_e_) (µg·g^−1^), pseudo-second order rate constant (k_2_) (g·mg^−1^·min^−1^), and fit accuracy using a pseudo-second-order kinetic plot for the adsorption of 16 polycyclic aromatic hydrocarbons (PAHs) onto (ZrO_2_)*_x_*(VPA) and (ZrO_2_)*_x_*(PPA) nanocomposites.

Adsorbent		(ZrO_2_)*_x_*VPA			(ZrO_2_)*_x_*PPA		
Zr/P Ratio	PAH Pollutant	q_e_	k_2_	R^2^	q_e_	k_2_	R^2^
50	Naphtalene	15.5	5	0.9971	17.5	6	0.9976
100		16.4	5	0.9902	16.8	15	0.9941
200		17.2	6	0.9902	18.5	2	0.9977
50	Acenaphthylene	14.7	10	0.9992	17.2	8	0.9968
100		15.7	8	0.9949	16.4	21	0.9961
200		17.2	7	0.9979	17.2	5	0.9903
50	Acenaphthene	17.5	11	0.9996	18.8	10	0.9952
100		17.9	11	0.9978	18.2	26	0.9981
200		18.7	7	0.9971	19.2	3	0.9954
50	Fluorene	17.4	16	0.9996	18.8	12	0.9966
100		18.1	6	0.9965	18.3	34	0.9991
200		18.7	9	0.9979	19	5	0.9973
50	Phenanthrene	17.2	29	0.9995	18.4	20	0.9979
100		17.6	13	0.9991	18	57	0.9994
200		18.4	12	0.9986	18.6	7	0.9982
50	Anthracene	18.3	33	0.9996	18.3	13	0.9971
100		17.2	12	0.9982	18.4	60	0.9997
200		18.6	12	0.9975	18.6	12	0.9992
50	Pyrene	17.9	67	0.9999	18.3	46	0.9993
100		18	27	0.9998	18.5	154	0.9999
200		18.5	25	0.9995	18.4	20	0.9996
50	Fluoranthene	17.7	51	0.9997	18.1	42	0.9992
100		17.8	25	0.9998	18.4	129	0.9998
200		18.4	21	0.9993	18.3	17	0.9993
50	Benz(a)anthracene	19.1	7	0.9971	18.9	59	0.9996
100		18.9	7	0.9966	19	136	0.9995
200		19.5	20	0.9999	15.5	9	0.9905
50	Chrysene	19.4	6	0.9961	19.1	148	0.9996
100		19.2	9	0.9941	19	81	0.9989
200		19.7	16	0.9999	13.5	10	0.9976
50	Benzo(b)fluoranthene	18.7	15	0.9992	18.8	118	0.9999
100		18.6	26	0.9996	18.6	228	0.9998
200		18.8	73	0.9953	16.9	35	0.9971
50	Benzo(k)fluoranthene	19.1	5	0.9949	18.8	98	0.9997
100		19.1	8	0.9901	18.8	54	0.9991
200		19.3	16	0.9999	14.3	10	0.9944
50	Benzo(a)pyrene	19.6	5	0.9944	19.3	139	0.9969
100		19.4	9	0.9935	19.2	78	0.9991
200		19.9	16	0.9999	14.3	10	0.9944
50	Dibenz(a,h)anththracene	19.8	4	0.9975	19.5	72	0.9983
100		18.9	10	0.9987	19.6	53	0.9965
200		20.3	9	0.9998	10.1	10	0.9157
50	Indeno(1,2,3-cd)pyrene	18.1	8	0.9978	17.8	140	0.9996
100		17.7	9	0.9994	17.9	89	0.9994
200		19.7	10	0.9979	15	21	0.9946
50	Benzo(ghi)perylene	18.6	5	0.9931	17.9	189	0.9997
100		18.4	15	0.9927	18.4	38	0.9987
200		18.9	18	0.9998	11.5	23	0.9618

## Data Availability

The data presented in the study can be requested from the authors.

## Appendix A

**Table A1:** Textural properties for (ZrO_2_)*_x_*(VPA) and (ZrO_2_)*_x_*(PPA) nanohybrids.

Adsorbent	S_BET_ (m^2^ g^−1^)
ZrO_2_	58
(ZrO_2_)_200_(VPA)	57
(ZrO_2_)_100_(VPA)	51
(ZrO_2_)_50_(VPA)	50
(ZrO_2_)_25_(VPA)	56
(ZrO_2_)_200_(PPA)	58
(ZrO_2_)_100_(PPA)	57
(ZrO_2_)_50_(PPA)	56
(ZrO_2_)_25_(PPA)	53

**Table A2:** Adsorption capacities (q_e_) (µg.g^−1^), pseudo-second order rate constant (k_2_) (g.mg^−1^.min^−1^), and fit accuracy using a Pseudo-second-order Kinetic Plots for the Adsorption of 16 PAHs onto (ZrO_2_) and (ZrO_2_)_50_(PPA) nanocomposites.

PAH	Log Kow *	ZrO_2_			(ZrO_2_)_50_(PPA)		
		q_e_ (mg.g^−1^)	k_2_ (g.mg^−1^ min^−1^)	R^²^	q_e_ (mg.g^−1^)	k_2_ (g.mg^−1^ min^−1^)	R^²^
Naphtalene	3.37	0.0087	35.8	0.9961	0.0077	38.5	0.9991
Acenaphtylene	4	0.0073	55.1	0.9903	0.0066	87.4	0.9996
Acenaphtene	3.92	0.0082	59.9	0.9954	0.0075	102.8	0.9997
Fluorene	4.18	0.0081	83.2	0.9957	0.0076	179.0	0.9997
Anthracene	4.54	0.0083	139.2	0.9967	0.0079	362.6	0.9998
Phenantrene	4.57	0.0088	264.4	0.9991	0.0087	450.9	0.9999
Fluoranthene	5.22	0.0088	258.8	0.9991	0.0087	330.7	0.9997
Pyrene	5.18	0.0088	255.0	0.9992	0.0086	258.6	0.9995
Benz(a)anthracene	5.91	0.0095	192.4	0.9999	0.0096	697.4	0.9999
Chrysene	5.91	0.0094	375.2	0.9999	0.0095	576.1	0.9999
Benzo(a)pyrene	5.91	0.0093	253.1	0.9999	0.0100	661.5	0.9999
Benzo(b)fluoranthene	5.8	0.0094	449.4	1.0000	0.0098	869.9	0.9999
Benzo(k)fluoranthene	6	0.0095	279.1	1.0000	0.0095	860.1	0.9999
Benzo(ghi)perylene	6.5	0.0095	161.1	0.9997	0.0098	1008.1	0.9999
Indeno(1.2.3-cd)pyrene	6.5	0.0093	396.9	0.9999	0.0098	1008.1	0.9999
Dibenz(a.h)anthracene	6.75	0.0094	493.4	0.9999	0.0099	1543.9	0.9999

* K_ow_ values are obtained from reference [37].

**Table A3:** Freundlich parameters, and fit accuracy using the slope of the linear plot of lnqe versus lnCe for the adsorption of 16 PAHs onto (ZrO_2_) and (ZrO_2_)_50_(PPA) nanocomposites.

PAH	Molecular weight (g/mol)	ZrO_2_			(ZrO_2_)_50_(PPA)		
		R^2^	K_F_	n_F_	R^2^	K_F_	n_F_
Naphtalene	128	0.996	2.94	0.92	0.961	4.68	0.33
Acenaphtylene	152	0.986	2.39	0.87	0.976	3.01	0.44
Acenaphtene	154	0.989	3.00	0.88	0.977	2.99	0.57
Fluorene	166	0.965	2.98	0.63	0.965	2.98	0.63
Anthracene	178	0.995	5.40	0.71	0.996	5.57	0.53
Phenantrene	178	0.997	4.72	0.69	0.971	4.21	0.59
Fluoranthene	202	0.999	4.75	0.76	0.946	5.42	0.52
Pyrene	202	0.999	5.37	0.72	0.986	6.25	0.53
Benz(a)anthracene	228	0.999	2.06	1.28	0.995	1.47	1.02
Chrysene	228	0.991	1.46	1.61	0.974	1.79	0.51
Benzo(a)pyrene	252	0.996	1.78	1.35	0.980	1.66	0.56
Benzo(b)fluoranthene	252	0.981	3.04	1.53	0.993	3.00	0.93
Benzo(k)fluoranthene	252	0.999	1.12	2.19	0.987	2.03	0.67
Benzo(ghi)perylene	276	0.955	1.31	1.55	0.985	1.52	0.75
Indeno(1.2.3-cd)pyrene	276	0.999	1.54	1.93	0.978	1.58	0.99
Dibenz(a.h)anthracene	278	0.941	1.84	1.00	0.941	2.29	0.48
